# Evaluation of the efficacy of Simparica Trio^®^ in the prevention of the transmission of *Babesia canis* by infected *Dermacentor reticulatus* to dogs

**DOI:** 10.1186/s13071-023-06115-2

**Published:** 2024-02-02

**Authors:** Stasia Borowski, Alta Viljoen, Lina D’Hanis, Sean Mahabir, Thomas Geurden

**Affiliations:** 1 Zoetis Belgium SA, Veterinary Medicine Research & Development, Mercuriusstraat 20, 1930 Zaventem, Belgium; 2 Clinvet International, Uitzich Road, Bainsvlei, Bloemfontein, 9338 South Africa; 3 Zoetis, Veterinary Medicine Research & Development, 333 Portage Street, Kalamazoo, MI 49007 USA

**Keywords:** Dogs, *Dermacentor reticulatus*, *Babesia canis*, Babesiosis, Transmission, Efficacy

## Abstract

**Background:**

*Babesia canis* is a clinically relevant vector-borne pathogen in dogs, and its presence is expanding. The efficacy of Simparica Trio^®^ (Zoetis) in the prevention of *B. canis* transmission was evaluated at the minimum recommended label dose of 1.2 mg/kg sarolaner, 24 µg/kg moxidectin and 5 mg/kg pyrantel per kg bodyweight.

**Methods:**

Twenty-four (24) dogs were randomly allocated to either a placebo-treated group or one of two treatment groups with Simparica Trio. Dogs were infested with *B. canis*-infected *Dermacentor reticulatus* ticks 21 or 28 days after treatment administration. Blood samples for antibody and DNA detection were collected from each dog prior to tick infestation until 28 days after infestation. A dog was defined as being *B. canis* positive if it tested positive by both an indirect immunofluorescence assay (IFA) and PCR at any time during the study.

**Results:**

No treatment-related adverse reactions were recorded during the study. All placebo-treated animals displayed clinical signs due to babesiosis and tested positive on both IFA and PCR. None of the Simparica Trio-treated animals displayed any clinical symptoms or tested positive, resulting in a 100% efficacy in the prevention of canine babesiosis (*P* < 0.0001).

**Conclusions:**

A single treatment with Simparica Trio at the minimum recommended label dose of 1.2 mg/kg sarolaner, 24 µg/kg moxidectin and 5 mg/kg pyrantel per kg bodyweight prevents the transmission of *B. canis* by infected *D. reticulatus* to dogs for at least 28 days.

**Graphical Abstract:**

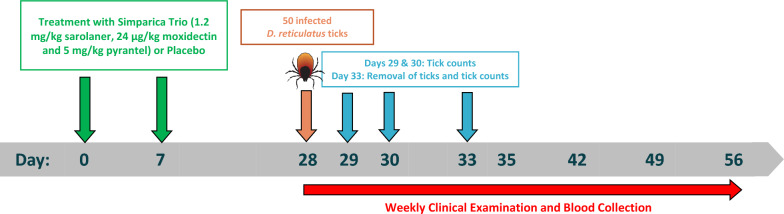

## Background

Many dogs are exposed to pathogens transmitted by ticks globally, including *Babesia canis*, which can cause babesiosis in dogs. Canine babesiosis can be associated with moderate illness or with more severe clinical findings including pale mucous membranes and icterus, anorexia, pyrexia and splenic enlargement. Infection with *B. canis* can also lead to mortality. In the European Union (EU), *B. canis* is mainly transmitted by adult *Dermacentor reticulatus* ticks [1, 2]. Due to climate change, increased international travel of pets as well as changes in land use and host distribution, its distribution is known to have expanded in Europe [3–7]. In parallel with the geographical expansion of its vector, *B. canis* is increasingly reported in new endemic foci, for example in the UK [8] and The Netherlands [9]. In addition, the total duration of tick questing activity in winter months has been reported to increase in several countries [10–14]. As a result, the prevalence of canine babesiosis increases in those regions where the tick is common [15], justifying the recommendation for year-round tick control measures in these areas [9]. The systemic isoxazolines are known to prevent the transmission of *B. canis* [19, 20, 22, 23] in addition to topically applied contact acaricides [16–18, 21]. The current study was conducted to evaluate the efficacy of a single treatment with Simparica Trio^®^ (Zoetis) at the minimum recommended label dose of 1.2 mg/kg sarolaner, 24 µg/kg moxidectin and 5 mg/kg pyrantel in the prevention of *B. canis* transmission when dogs are challenged with infected *D. reticulatus* ticks 21 and 28 days after treatment.

## Materials and methods

### Animals

In total, 24 laboratory animals (13 males and 11 females, originated from the study site’s colony) were enrolled in this study. These animals were either Beagles or mongrels and were between 2 and 7.5 years of age and weighed between 11.8 and 26.4 kg at the start of the study. All dogs were found healthy and clinically normal by the examining veterinarian during the physical examination at the start of the study. All dogs were vaccinated and dewormed at least 14 days prior to first treatment administration and had undergone a wash-out period to ensure that no residual ectoparasiticide efficacy remained from any previously administered compounds. Dogs were confirmed to be free of ticks and seronegative for *B. canis* prior to enrollment. Dogs were housed individually but had auditory and visual contact to the dogs on the opposite side of the individual housing. The animal enclosures were indoors with each pen containing one resting area. Social interactions with the dogs were performed daily for at least 2 min per dog. Dogs were given an appropriate commercial dog food once daily, and water was provided from bowls and replenished at least twice daily. The general health of the dogs was observed at least twice daily.

### Treatment administration

Dogs were randomly allocated to treatment groups, rooms and pens according to a randomized block design with one-way treatment structure within room. Blocking was based on pen location and pre-treatment body weight within room. Dogs in treatment group T01 received a placebo tablet on Days 0 and 7. Dogs in treatment group T02 were administered Simparica Trio on Day 0 and placebo on Day 7 to evaluate efficacy 28 days after treatment. Dogs in treatment group T03 received placebo on Day 0 and Simparica Trio on Day 7 to evaluate efficacy 21 days after treatment. The formulation of the placebo was identical to Simparica Trio but without active ingredients. Dogs were administered a single tablet or a combination of tablets to achieve the minimum label dose of 1.2 mg/kg sarolaner, 24 µg/kg moxidectin and 5 mg/kg pyrantel. Treatments were administered orally. Feed was withdrawn the day before treatment and offered again ≥ 4 h after treatment administration. Dogs were observed immediately after dosing for evidence that the dose was swallowed and for potential adverse events and at approximately 2 h post dosing for evidence of vomiting. Clinical observations were performed on Days 0 and 7 prior to and at approximately 1, 3, 6 and 24 h after treatment administration. Masking was accomplished by separation of functions of study personnel. All persons making observations, conducting tick infestations and counts, sampling or performing laboratory analyses were masked to treatment allocation.

### Tick infestation and tick counts

On Day 28, each dog was infested with 50 (± 4) viable, unfed adult *D. reticulatus* ticks in a 1:1 sex ratio. A laboratory-bred *D. reticulatus* tick strain infected with *B. canis* was used, with the tick strain originating from BLE laboratories in Ireland and the pathogen derived from a clinical case of canine babesiosis in The Netherlands. The infection rate with *B. canis* was determined for a random sample of 50 ticks by PCR analysis and was 22.4%, which was considered an adequate infection pressure. Ticks were applied directly on the dog by tapping the vial to dislodge the ticks from the container so that they could be spread directly over the dog’s hair coat. Dogs were restrained for 10 min and confined in an infestation chamber to enhance tick attachment for approximately 4 h. Ticks dislodged during the first 10 min were placed back on the dog. At 24 (± 4) and 48 (± 4) h after infestation, each dog was examined visually and by hand to count the numbers of live and dead, attached and unattached ticks. On Day 33, ticks were again counted and categorized as above and removed. The engorgement status was determined for the collected attached ticks.

### Clinical evaluation of babesiosis

Clinical examination for signs of babesiosis were conducted for all dogs on Days 28 (prior to tick infestation), 35, 42, 49 and 56 and included determination of rectal temperature, body weight, respiratory rate and heart rate, and observations for the presence of lethargy, anorexia, lymph node enlargement, ataxia, pale mucous membranes, pigmenturia, icterus and other potential signs of babesiosis (e.g. petechia, ecchymosis, hyphema, lameness). Pigmenturia was assessed by the colour of the urine (which could easily be observed on the light epoxy floors of the animal pens).

### Blood sampling and analysis

Blood samples were collected from each dog on Day 28 (prior to tick infestation) for *B. canis* antibody assay and on Days 35, 42, 49 and 56 for *B. canis* antibody assay and PCR analysis. Blood samples were centrifuged and serum frozen until tested for *B. canis* antibodies using an indirect immunofluorescence assay (IFA) performed by Clinomics (MegaFLUO^®^ BABESIA canis commercial test kit with *B. canis* antigen-coated slides). Total genomic DNA was isolated from the whole blood samples using a commercial genomic DNA isolation kit (GeneJet Genomic DNA Purification Kit, Catalog number: K0721). PCR entailed the use of primers specific to the *B. canis* internal transcribed spacer region of the DNA. A PCR product of approximately 300 bp indicated the presence of the target region in the sample. Positive, negative, no template as well as internal amplification controls were included in each run. If a dog displayed an abnormally high body temperature (> 39.4 °C) and/or showed clinical symptoms associated with babesiosis, two blood smears were prepared and examined for the presence of *B. canis*. If the dog was diagnosed positive for babesiosis on a blood smear, blood for PCR analysis was collected from the dog prior to being rescue-treated.

### Efficacy evaluation

The experimental unit for treatment was the animal. The efficacy of Simparica Trio in the prevention of *B. canis* transmission by *D. reticulatus* ticks was calculated based on the proportion of ‘ever positive’ dogs in the treated groups compared to the control group. A dog was defined as being ‘ever positive’ for *B. canis* if the dog tested positive by IFA and PCR at any time after infestation. The infection rate for each treatment group was calculated as the proportion of ‘ever positive’ dogs in the respective treatment group. A Fisher’s exact test was used to test for the overall treatment effect and to compare the infection rate for the treated groups to that of the control group.

Efficacy (%) = 100 x (Tc–Tt)/Tc, where Tc = Total number of infected dogs in the negative control group T01 and Tt = Total number of infected dogs in the treatment group T02 or T03.

The acaricidal efficacy was calculated based on the percent reduction of arithmetic mean live tick counts (attached and free) in the treated groups compared to the control group using Abbott’s formula:

$$\% \, reduction\, = \,100 \, \times \, \frac{{{\text{mean }}\,{\text{count }}\,\left( {{\text{placebo}}} \right)\,{ }{-}\,{\text{ mean}}\,{\text{ count}}\,{ }\left( {{\text{treated}}} \right)}}{{{\text{mean}}\,{\text{ count }}\,\left( {{\text{placebo}}} \right)}}$$

Live tick counts without removal were analyzed using a general linear mixed model for repeated measures. The model included the fixed effect of treatment, time point and the treatment by time point interaction. The random effects included room, block within room, the interaction between block and treatment within room (animal term) and error. Live tick counts with removal were analyzed using a general linear mixed model. The model included the fixed effect of treatment and the random effects of block and error. Least squares means and standard errors were calculated, and 95% confidence intervals were constructed by treatment and time point. A priori contrasts were used to assess pairwise comparisons between treatments within timepoint. As significant related treatment effects were present, treatment differences were assessed at each time point. All tests were done at the two-tailed 5% level of significance (*P* ≤ 0.05*).*

## Results

All 24 animals were dosed completely. No tablets were expelled, and no evidence of vomiting was observed in any animal up to 2 h after treatment. Prior to infestation with the ticks on Day 28, all animals were negative for the presence of *B. canis* antibodies (IFA). In the placebo-treated group, all eight dogs were found PCR positive 7 days after infestation with *D. reticulatus*. By 14 days after infestation, 5 out of 8 dogs were IFA positive, and all dogs were IFA positive 28 days after infestation. All placebo-treated dogs displayed at least one clinical sign of babesiosis. The clinical signs observed were lethargy (6 out of 8 animals), pigmenturia (3 out of 8 animals), pale mucous membranes (4 out of 8 animals), inappetence (1 out of 8 animals) and fever (> 39.4 °C, 6 out of 8 animals). All placebo-treated dogs were rescue-treated after being diagnosed with babesiosis. All dogs received Diminazine (Berenil RTU^®^, SC, once on the day of diagnosis), Kyro B + Liver (SC, once on the day of diagnosis), prednisolone acetate (Prednisolone 1%, SC, for 2 days), imidocarb diproprionate (Forray 65^®^, once on the day after diagnosis) and Hills A/D (twice a day for 3 days). One dog required additional treatment with Catosol (IV), dextrose 50% (IV) and Ringer's lactate (IV) 2 days after diagnosis. All dogs recovered well. None of the Simparica Trio-treated dogs tested positive by PCR or IFA at any time point. In treatment group T02, one dog had increased rectal temperature (39.5–39.8 °C) on Days − 8, 28, 35, 49 and 56 but the blood smears collected on Days 35, 49 and 56 were all negative for *B. canis*. The increased rectal temperature was likely due to the excitement of the dog given the repeated nature of the observation and the absence of any indication for infection. In treatment group T03, one dog had increased rectal temperature (39.5–40.2 °C) on Days − 8, 28 and Days 34 until 36. A blood smear was collected on Days 34, 35 and 36, which were negative for *B. canis*. Lethargy, pigmenturia, pale mucous membranes or inappetence (clinical signs indicating babesiosis) was not noted in either of these two animals. As none of the Simparica Trio-treated dogs were ever positive for *B. canis* infection, this indicated 100% efficacy (*P* < 0.0001) in the prevention of *B. canis* infection in dogs exposed to infected ticks for at least 28 days after treatment with Simparica Trio (Table 1). The acaricidal efficacy was 73.3 and 81.0% on Day 29 (24 h after infestation) in T02 and T03 respectively. On Day 30 (48 h after infestation), the percent reduction was 99.5% in T03 and 100% in T02. On Day 33, no live ticks were found on any of the treated dogs so the acaricidal efficacy was 100% in both treatment groups. The *D. reticulatus* tick counts were significantly lower (*P* < 0.0001) in both Simparica Trio-treated groups compared to the placebo-treated group on all counting days (Table 2).

**Table 1:** Number (and percentage) of dogs positive for *Babesia canis* on blood smear, PCR and IFA and number (and percentage) of dogs found to be ever positive as well as the percent efficacy of Simparica Trio^®^ in the prevention of transmission of *B. canis*

Treatment	Number (percentage) of animals positive for *B. canis*				Percent efficacy^c^
	Blood smear^a^	PCR	IFA	Ever positive^b^	
T01 (Placebo)	8 of 8 (100%)	8 of 8 (100%)	8 of 8 (100%)	8 of 8 (100%)	N/A
T02 (Simparica Trio 28 days before infestation)	0 of 1 (0%)	0 of 8 (0%)	0 of 8 (0%)	0 of 8 (0%)*	100%
T03 (Simparica Trio 21 days before infestation)	0 of 1 (0%)	0 of 8 (0%)	0 of 8 (0%)	0 of 8 (0%)*	100%

*PCR* polymerase chain reaction, *IFA* indirect immunofluorescence assay

^a^Blood smears were collected from dogs with rectal temperature > 39.4 °C or clinical symptoms associated with babesiosis

^b^A dog was ever positive for *B. canis* if it had positive results for both IFA and PCR test

^c^Percent efficacy for T0X = 100 × (number infected in T01—number infected in T0X)/number infected in T01, where *X* = 2, 3

^*^Number of ‘ever positive’ dogs significantly lower than in T01 (*P* < 0.0001)

**Table 2:** Live *Dermacentor reticulatus* counts at 1, 2 and 5 days after infestation: arithmetic mean, least squares mean, range and percent reduction compared to the control group and statistical comparisons

Days after infestation	Treatment^a^	Arithmetic mean	Least squares mean	Range	Percent reduction^b^	Control *vs* T0X *P*-value
1	T01	27.6	27.6	12–43	Na	
	T02	7.4	7.4	3–21	73.3	< 0.0001
	T03	5.3	5.3	0–18	81.0	< 0.0001
2	T01	27.4	27.4	17–38	Na	
	T02	0.0	0.0	0–0	100.0	< 0.0001
	T03	0.1	0.1	0–1	99.5	< 0.0001
5	T01	34.4	34.4	29–39	Na	
	T02	0.0	0.0	0–0	100.0	< 0.0001
	T03	0.0	0.0	0–0	100.0	< 0.0001

*Na* not applicable

^a^T01: dogs received placebo, T02: dogs received Simparica Trio 28 days before infestation, T03: dogs received Simparica Trio 21 days before infestation

^b^Calculated using the formula [(C-T)/C] × 100, where *C* = arithmetic mean of live tick counts for the control group and *T* = arithmetic mean of live ticks counts for the treated group

## Discussion

The *B. canis* infection model was previously used [18–24], and as in previous studies [16, 20–24], a dog was considered as *B. canis* positive if both IFA and PCR testing indicated infection. As in the study evaluating the efficacy of Simparica^®^ [22], a high number (*n* = 50) of ticks with a high (22.4%) *B. canis* infection rate was used in the current study, which corresponds to reported *B. canis* prevalences in *D. reticulatus* ticks in the field [25–27]. This severe challenge, the evaluation of the efficacy towards the end of the treatment period (21 and 28 days) and the treatment at the minimum recommended label dose therefore allowed to evaluate a worst case scenario for *B. canis* transmission in the current study. Indeed, all placebo-treated animals in the current study developed clinical symptoms within 1 week after tick infestation. While all positive dogs were immediately treated after they displayed clinical symptoms and tested positive by blood smear, the treatment did not impede the PCR diagnosis as the blood samples of all placebo-treated animals yielded an amplification product 7 days after the infestation. Furthermore, all placebo-treated animals seroconverted within 28 days after infestation.

The prevention of pathogen transmission through tick feeding has become an important characteristic for acaricidal products. In a previous study, the efficacy of sarolaner (Simparica) in the prevention of babesiosis in dogs was already demonstrated [22], and the current study confirms the high efficacy of Simparica Trio at the minimum dose 1.2 mg/kg sarolaner, 24 µg/kg moxidectin and 5 mg/kg pyrantel per kg bodyweight for at least 28 days. Simparica Trio has rapid and consistent efficacy against ticks with persistent efficacy of at least 4 weeks after a single treatment at the minimum recommended label dose [28]. The acaricidal efficacy of sarolaner against *D. reticulatus* was confirmed in the present study and indeed resulted in the prevention of *B. canis* transmission at the end of the monthly dosing interval.

## Conclusions

The current study confirms the rapid and consistent acaricidal efficacy of Simparica Trio against *D. reticulatus* and its ability to prevent canine babesiosis caused by *B. canis* under high challenge conditions and at the minimum recommended label dose. The persistent efficacy and rapid speed of kill for at least 4 weeks provided by Simparica Trio provide a continued protection against tick infestation and against pathogen transmission in a monthly treatment regime.

## Data Availability

Data upon which the conclusions are based have been presented in the article.

## Abbreviations

IFA: Indirect immunofluorescence assay
IV: Intravenous
PCR: Polymerase chain reaction
SC: Subcutaneous
